# Amyloid β-dependent mitochondrial toxicity in mouse microglia requires P2X7 receptor expression and is prevented by nimodipine

**DOI:** 10.1038/s41598-019-42931-2

**Published:** 2019-04-24

**Authors:** Paola Chiozzi, Alba Clara Sarti, Juana M. Sanz, Anna Lisa Giuliani, Elena Adinolfi, Valentina Vultaggio-Poma, Simonetta Falzoni, Francesco Di Virgilio

**Affiliations:** 1 Department of Morphology, Surgery and Experimental Medicine, University of Ferrara, Ferrara, Italy; 2 Department of Medical Sciences, University of Ferrara, Ferrara, Italy

**Keywords:** Neuroimmunology, Chronic inflammation, Inflammasome

## Abstract

Previous data from our laboratory show that expression of the P2X7 receptor (P2X7R) is needed for amyloid β (Aβ)-stimulated microglia activation and IL-1β release *in vitro* and *in vivo*. We also showed that Aβ-dependent stimulation is inhibited by the dihydropyridine nimodipine at an intracellular site distal to the P2X7R. In the present study, we used the N13 microglia cell line and mouse primary microglia from wt and *P2rx7*-deleted mice to test the effect of nimodipine on amyloid β (Aβ)-dependent NLRP3 inflammasome expression and function, and on mitochondrial energy metabolism. Our data show that in microglia Aβ causes P2X7R-dependent a) NFκB activation; b) NLRP3 inflammasome expression and function; c) mitochondria toxicity; and these changes are fully inhibited by nimodipine. Our study shows that nimodipine is a powerful blocker of cell damage caused by monomeric and oligomeric Aβ, points to the mitochondria as a crucial target, and underlines the permissive role of the P2X7R.

## Introduction

Alzheimer’ s disease (AD), despite a slight decrease in incidence in developed countries over the last twenty years, is still a leading cause of dementia world-wide^1^. About 95% of AD cases are sporadic and occur in patients over 60 years of age (late-onset AD), while about 5% are familial, and occur at a much earlier age (even at 30 years)^1^. Familial AD shows autosomal dominance in at least one of three main genes: amyloid precursor protein, Presenilin 1 or Presenilin 2^2^. The histopathological hallmark is deposition of aggregates of amyloid β (Aβ) peptides in the extracellular space, and intracellular accumulation of neurofibrillary tangles^2^. These extracellular and intracellular changes are thought to cause progressive synaptic deficit and neuronal death. The pathogenic mechanism responsible for Aβ-dependent neurodegeneration is largely obscure albeit it is increasingly recognized that Aβ-driven inflammation has a leading role^3^. Several studies in the past have stressed the pathogenic relevance of Aβ-stimulated TNFα, IL-1β or reactive oxygen species (ROS) accumulation in AD brains and the ensuing neuroinflammation^4–6^. Without doubt, the main player in neuroinflammation is microglia, with a dual role, as a protective factor responsible for clearance of extracellular Aβ-fibrils, or as an injurious agent responsible for release of neurotoxic factors, in line with the known Dr. Jekyll/Mr. Hyde role of inflammation itself^7,8^. Acutely, Aβ triggers release of inflammatory mediators from microglia, but on the long run such sustained stimulation causes an irreversible damage^8,9^.

The likely inflammatory pathogenesis of AD calls for the search for novel anti-inflammatory agents active across the blood-brain-barrier (BBB), but re-evaluation of older, maybe less trendy, but no doubt widely tested drugs is also warranted. Dihydropyridines are among these re-discovered drugs. Dihydropyridines are L-type calcium blockers permeable across the BBB, and widely used to treat hypertension. It has been suggested that nimodipine and nifedipine might be beneficial to increase brain blood flow, and therefore alleviate cognitive impairment in AD^10^, but these observations had little or no clinical follow-up. *In vitro* experiments showed a protective effect in neuron/microglia co-cultures, an effect putatively assigned to inhibition of TNFα and IL-1β release^11^. The protective effect of dihydropyridines might be due to inhibition of L-type voltage-dependent Ca^2+^ channels^12,13^ or to blockade of an unrelated intracellular pathway (see also ref. ^14^). In our previous study, we observed that the massive plasma membrane depolarization caused by opening of the P2X7R in N13 microglia was not paralleled by a nimodipine-sensitive Ca^2+^ uptake, thus suggesting that L-type Ca^2+^ channels did not play a major role in P2X7R-dependent activation of this cell type^9^. Thus we postulated that the inhibitory activity of nimodipine was mainly due to off-target effects. Our previous *in vitro* data showed that nimodipine (and nifedipine) are powerful inhibitors of intracellular pro-IL-1β accumulation as well as pro-IL-1β cleavage and mature Il-1β release induced by Aβ or by extracellular ATP in microglia. Very interestingly, *in vivo* nimodipine administration, at concentrations know to be routinely reached in the CNS during therapeutical administration, significantly reduced IL-1β accumulation triggered by intra-hippocampal Aβ inoculation^9^. Based on these data we hypothesized that nimodipine targeted one or more intracellular pathways involved in the activation of microglia. We also previously showed that the P2X7 receptor (P2X7R) has a key role in Aβ-dependent microglial activation and injury^9,15^. The P2X7R is a widely distributed ATP-gated plasma membrane ion channel that is receiving increasing attention as pro-inflammatory molecule associated to cytokine release and T lymphocyte differentiation^16^. More recently, the possible P2X7R role in AD has been highlighted^17^. Although it was initially thought that pathogenic activity was mainly associated to fibrillar Aβ, it is now clear that soluble Aβ is also toxic^18,19^, therefore the effect of the monomeric or oligomeric Aβ is worth of investigation.

In the present study, we investigate the effect of soluble Aβ on mouse microglia and show that nimodipine not only inhibits Aβ-dependent NFκB and NLRP3 inflammasome stimulation, but also prevents Aβ-mediated mitochondrial damage. In addition, we show that in microglia expression of the P2X7R is an absolute requirement for Aβ pro-inflammatory activity and mitochondrial toxicity. In conclusion, our study stresses the role of the P2X7R in Aβ-dependent microglial cell damage, shows that mitochondria are a crucial target and highlights the potential benefit of nimodipine as therapeutic agent.

## Results

**Figure 1:**
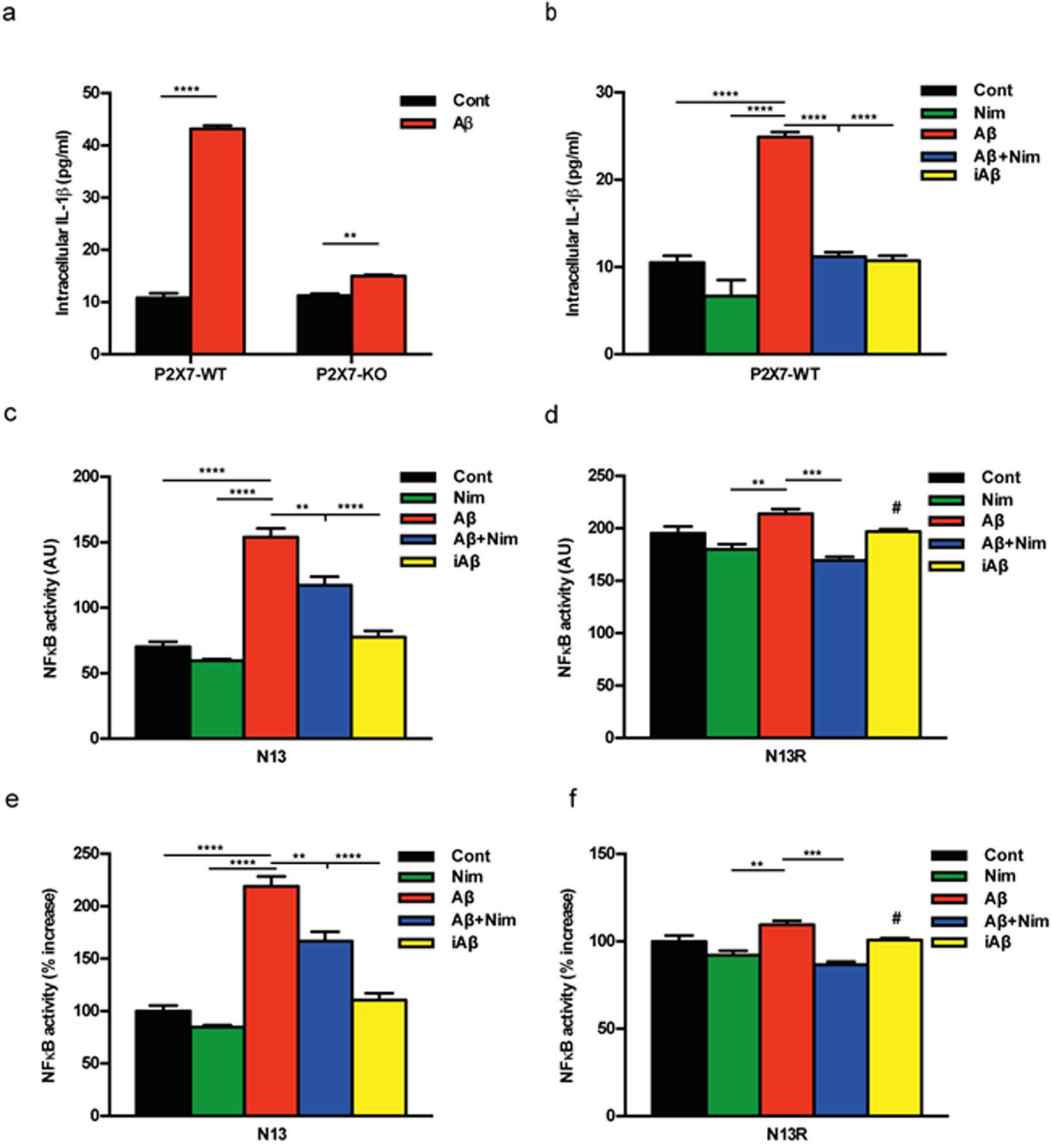
The P2X7R is an absolute requirement for Aβ-dependent microglia activation. Primary mouse microglia (**a**,**b**), suspended in 200 µl of FCS-supplemented astrocyte-conditioned medium was plated in 48-well culture dishes at a concentration of 2 × 10^4^/well, and stimulated for 24 h at 37 °C with the various agents as indicated. At the end, cells were rinsed, suspended in FCS-free RPMI, lysed and analysed for IL-1β content (**a**,**b**). For NFκB measurement, wt N13 or N13R cells were seeded in 10-cm Petri dishes, at a concentration of 10^6^/dish, treated for 24 h at 37 °C with the various stimuli, and processed as described in Methods (**c–f**). Stimulant concentration was: Aβ, 4 μM; iAβ, 4 μM; nimodipine (nim), 36 nM. Data are means ± SEM from 3 to 5 independent experiments, each performed in triplicate, for a total of 9 to 15 individual determinations. **p < 0.01; ***p < 0.001; ****p < 0.0001; # not significant versus controls.

**Figure 2:**
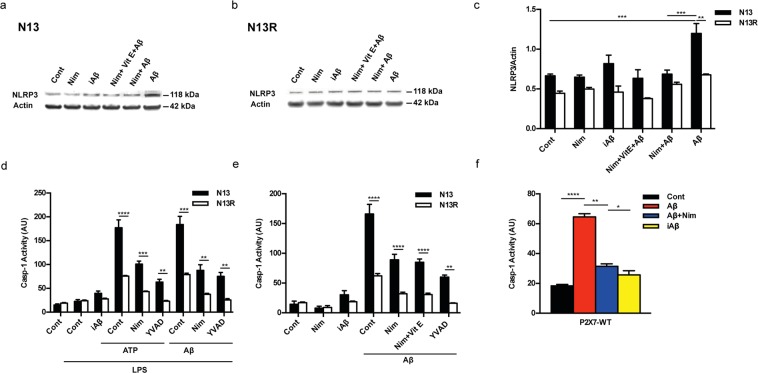
The P2X7R is needed for Aβ-mediated activation of the NLRP3 inflammasome: inhibition by nimodipine. (**a**–**e**) wt N13 and N13R cells were suspended in 300 µl of FCS-supplemented RPMI medium, plated in 24 well culture dishes at a concentration of 1.5 × 10^4^/well. (**f**) Primary microglia was suspended in astrocyte-conditioned medium and processed as for N13 cells. In (**a**,**b**,**e**,**f**) cells were incubated for 24 h in the continuous presence of the various stimulants, while in **d** incubation was carried for a total of 5.5 h. Densitometric analysis of NLRP3 protein expression (**c**). ATP- and Aβ-stimulated casp-1 activation in the presence (**d**) and absence (**e**) of LPS pre-treatment. Effect of nimodipine on casp-1 activation in primary microglia (**f**). YVAD was 50 μM, Vit E 50 μM, ATP 3 mM; concentration of other stimulants was as in Fig. 1, except in panel d, where Aβ and iAβ were 10 μM. Cells were pretreated with LPS in complete RPMI (wt N13 and N13R cells), after 4 h cells were rinsed and suspended in FCS-free RPMI; where indicated, nimodipine or YVAD were added, and after 1 h of incubation, cells were challenged with iAβ, Aβ or ATP, then stimulated with either ATP or Aβ for 30 min. YVAD, Vit E, nimodipine (nim) or nimodipine plus Vit E were added 1 h before the addition of ATP or Aβ, whether in the presence or absence of LPS treatment. Data are means ± SEM from 3 separate experiments each performed in triplicate, for a total of 9 individual determinations. *p < 0.05; **p < 0.01; ***p < 0.001; ****p < 0.0001.

Amyloid β, but not the scrambled Aβ peptide (iAβ) is a powerful stimulus for intracellular accumulation of IL-1β in mouse microglia^15^. This effect is largely dependent on the expression of the P2X7R since it was strongly reduced in mouse microglia isolated from *P2rx7*-deleted mice (Fig. 1a). As previously shown by our laboratory^9^, pre-incubation with nimodipine inhibited Aβ-dependent release of mature IL-1β as well as intracellular IL-β accumulation (Fig. 1b and Supplementary Fig. S1). The main stimulus for pro-IL-1β accumulation is activation of the transcription factor NFκB. Effect of nimodipine on NFκB activation was tested in N13 mouse microglia, a cell line that can be easily grown to make available a sufficient amount of intracellular substrates for NFκB measurements. N13 cells are available as the wt and the P2X7R-low variant, selected and fully characterized in our laboratory for reduced P2X7R expression^20^. This variant is referred to as N13R, where R stands for “ATP-resistant”. Figure 1c shows that in wt N13 microglia Aβ, but not iAβ, caused a strong activation of NFκB that was significantly reduced by nimodipine. Intriguingly, we observed that levels of activated NFκB were about 3 folds higher in N13R than in wt N13 microglia, and were significantly reduced by nimodipine (Fig. 1d). Nimodipine also antagonized the small increase caused by Aβ in N13R cells. Addition of iAβ had no effect. These same data are shown in Fig. 1e,f as percent increase over resting levels in wt N13 and N13R cells, respectively. Besides nimodipine, other dihydropyridines such as nifedipine and nitrendipine also inhibited Aβ-stimulated IL-1β release (Supplementary Fig. S2). To verify whether dihydropyridine effect might be due to inhibition of voltage-gated Ca^2+^ channels reported to be expressed in mouse microglia^12,13^, we checked the effect on intracellular Ca^2+^ of plasma membrane depolarization. As shown in Supplementary Fig. S3, addition of KCl to N13 microglia caused no increases in the intracellular Ca^2+^ concentration, suggesting that this cell line expressed no functional voltage-gated Ca^2+^ channels. Besides causing accumulation of intracellular IL-1β, Aβ is also a potent stimulus for pro-IL-1β cleavage and secretion of the mature form, both effects being effectively inhibited by nimodipine^9^. Pro-IL-1β cleavage and mature IL-1β secretion are independent of NFκB activation, and due to stimulation of the NLRP3 inflammasome^21^. Thus, we checked the effect of Aβ and nimodipine on NLRP3 inflammasome. Figure 2a–c show that Aβ, but not iAβ, caused an increase in NLRP3 protein expression in wt N13 but not in N13R cells. Enhanced expression of NLRP3 was inhibited by nimodipine, alone or in combination with an anti-oxidant such as vitamin E (Vit E). Levels of the coupling factor apoptosis-associated speck-like protein containing a CARD (ASC) were low in both wt N13 and N13R under resting conditions, were not further increased by Aβ stimulation, and nimodipine had no effect under all conditions tested (not shown). Alongside with NLRP3 expression, Aβ also triggered caspase-1 (casp-1) activation in wt N13 cells, whether stimulated according to the standard protocol involving LPS priming (Fig. 2d), or in the absence of LPS priming (Fig. 2e). Of note, LPS alone had no effect on casp-1 activation (Fig. 2d). Aβ caused a stimulation of casp-1 similar to that triggered by ATP, a strong agonist for casp-1 activation. Nimodipine largely inhibited casp-1 activation, in both Aβ- and ATP-stimulated cells, to a level comparable to that of the canonical casp-1 blocker YVAD (Fig. 2d). N13R cells were much less sensitive to casp-1 stimulation by Aβ or by ATP in all conditions tested, but also in these cells nimodipine significantly decreased casp-1 activation. As shown in Fig. 2e, Aβ was also a potent stimulus for casp-1 activation in the absence of LPS-priming, and its effects were largely inhibited by nimodipine. Association of nimodipine with an anti-oxidant such as Vit E did not further inhibit casp-1 activity. Nimodipine also inhibited Aβ-stimulated casp-1 activation in primary microglia (Fig. 2f).

At variance with the massive inhibitory effect on IL-1β secretion, nimodipine had no effect on TNFα secretion stimulated by LPS and Aβ (Supplementary Fig S4).

**Figure 3:**
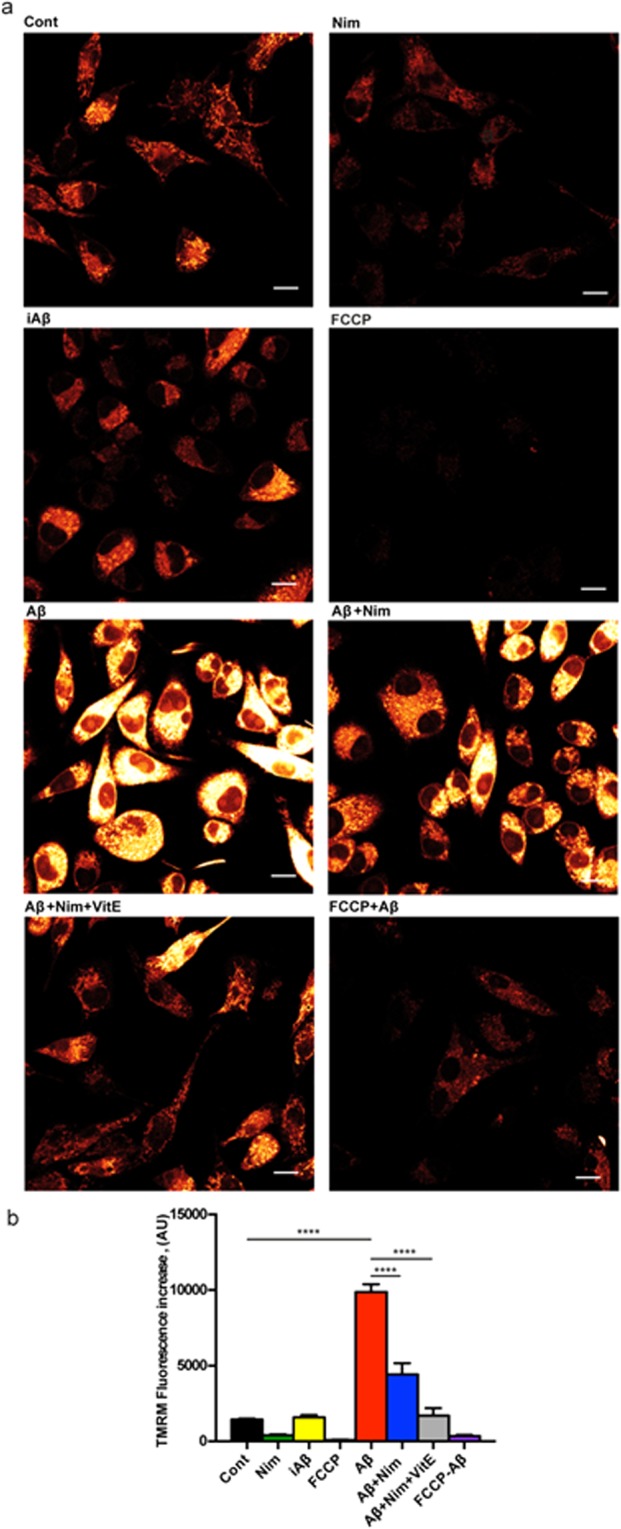
Aβ drives mitochondrial hyperpolarization in wt N13 microglia: inhibition by nimodipine. Microglia cells were suspended in 1 mL of FCS-supplemented RPMI medium at a concentration of 10^5^/mL, and seeded onto glass coverslip slides at 37 °C for 24 h; at the end of this incubation, cells were rinsed, suspended in FCS-free RPMI and the positively charged, potential sensitive dye TMRM was added. Dishes were analyzed at confocal microscopy and single cell fluorescence was acquired (see Methods). FCCP concentration was 1 µM; other stimulants concentration as in Figs 1 and 2. (**a**) Confocal microscopy pictures; bar = 10 μm. (**b**) Mean fluorescence emission ± SEM from 5 separate experiments each performed in triplicate for a total of 15 individual determinations. ****p < 0.000.1.

**Figure 4:**
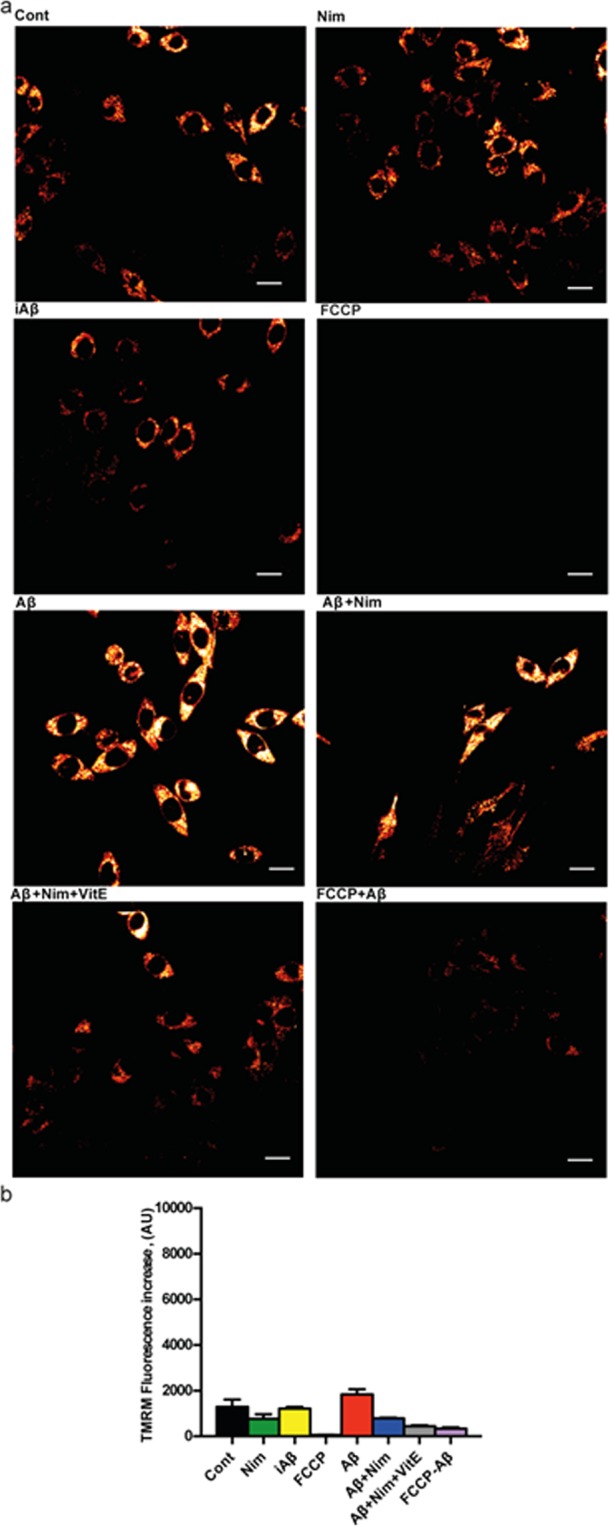
Aβ does not trigger mitochondrial hyperpolarization in N13R microglia. Microglia cells were suspended in 1 mL of FCS-supplemented RPMI medium and processed as described in Fig. 3. Agonist concentration and other experimental details as in Fig. 3. (**a**) Confocal microscopy pictures; bar = 15 μm. (**b**) Mean fluorescence emission ± SEM from 3 independent experiments, each performed in triplicate for a total of 9 individual determinations_._

**Figure 5:**
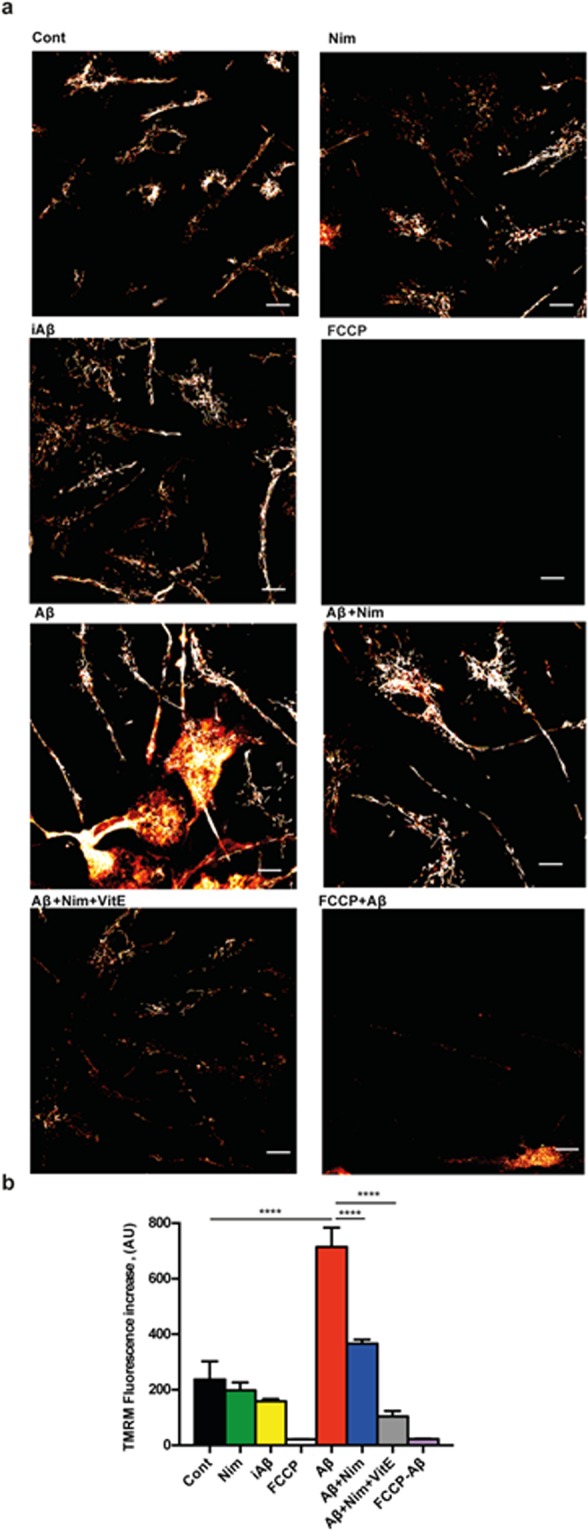
Aβ drives mitochondrial hyperpolarization in primary wt microglia: inhibition by nimodipine. Microglia cells were suspended in 1 mL of astrocyte-conditioned medium, plated at a concentration of 5 × 10^4^/mL, seeded onto glass coverslip slides at 37 °C for 24 h and processed as described in Fig. 3. Stimulant concentration as in Fig. 3. Bar = 10 μm. _(_**a**) Confocal microscopy pictures. (**b**) Mean fluorescence emission ± SEM from 3 independent experiments, each performed in triplicate for a total of 9 individual determinations. ****p < 0.0001.

**Figure 6:**
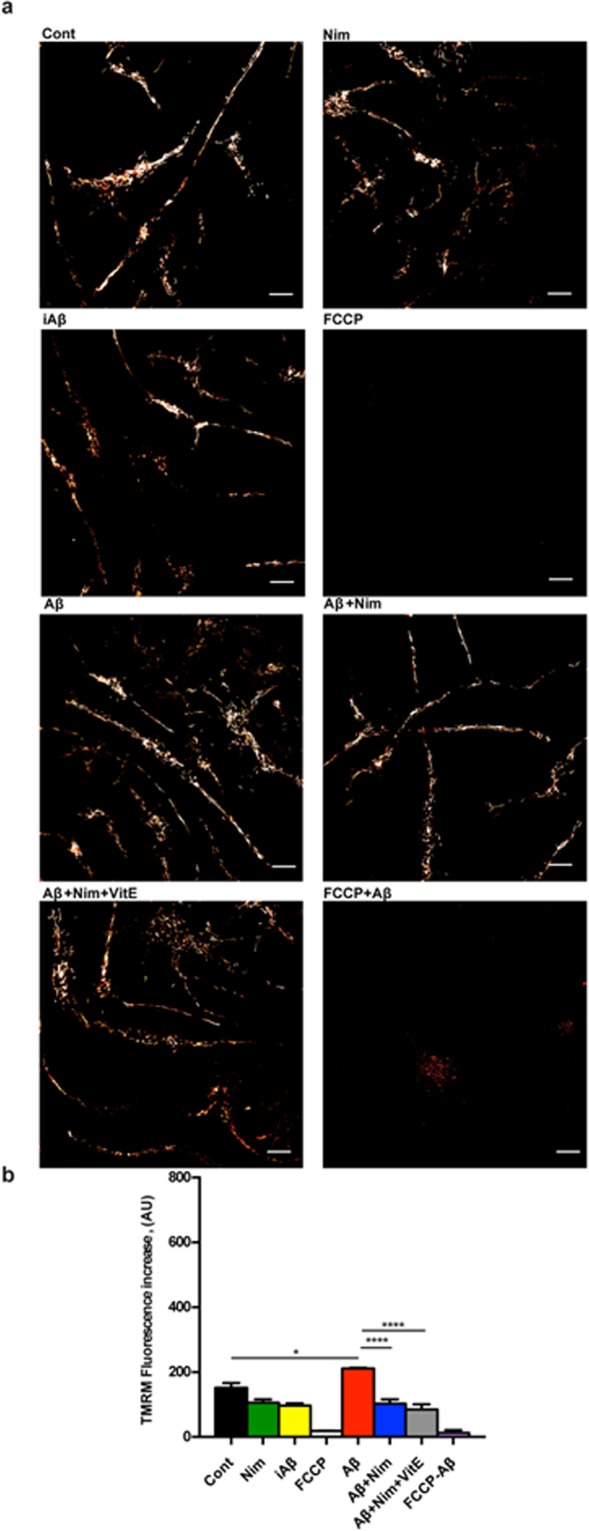
Aβ drives a reduced mitochondrial hyperpolarization in P2X7R-deleted primary microglia. Microglia cells were suspended in 1 mL of astrocyte-conditioned medium, plated at a concentration of 5 × 10^4^/mL on glass coverslip slides at 37 °C for 24 h, and processed as in Fig. 3. Stimulant concentration as in Fig. 3. (**a**) Confocal microscopy pictures; bar = 10 μm. (**b**) Mean fluorescence emission ± SEM from 3 independent experiments, each performed in triplicate for a total of 9 individual determinations. *p < 0.05; ****p < 0.0001.

The main pathway leading to NLRP3 inflammasome activation is the fast decrease in the cytosolic K^+^ concentration caused by opening of large conductance plasma membrane channels such as pannexin-1, connexins or the P2X7R itself^16,22^. However, it is unlikely that nimodipine hampers NLRP3 inflammasome activation by interfering with plasma membrane ion fluxes, as we previously showed that plasma membrane potential changes and Ca^2+^ fluxes in microglia were not affected by nimodipine^9^. Another known stimulus for NLRP3 inflammasome activation is mitochondrial generation of reactive oxygen species (ROS)^23^. Therefore, we investigated whether nimodipine might prevent NLRP3 inflammasome activation by interfering with Aβ-dependent changes of mitochondrial energy metabolism and ROS generation. Aβ is known to cause mitochondrial toxicity witnessed by plasma membrane potential collapse, fragmentation of the mitochondrial network and overall decrease of ATP synthetic activity^24^. In the experiments shown in Fig. 3a,b, N13 microglial cells were incubated in the presence of Aβ for 24 h, then rinsed and loaded with the mitochondrial potential sensitive dye tetramethylrodamine methylesther (TMRM) to stain the mitochondrial network. Our anticipation was that Aβ should cause a mitochondrial potential collapse, but to our surprise, Aβ caused instead a large mitochondrial potential increase that was fully inhibited by the un-coupler FCCP, thus validating the specific mitochondrial localization of the dye. The scrambled iAβ peptide was fully inactive. The Aβ-stimulated potential increase was prevented by pre-treatment with nimodipine. Basal mitochondrial potential in quiescent cells was also slightly reduced by nimodipine. Nimodipine effect was potentiated by the anti-oxidant Vit E. We previously reported that lack of the P2X7R protects mouse microglial cells from the injurious effect of Aβ^15^. Figure 4a,b shows that N13R cells had a lower mitochondrial potential, as previously reported in cells lacking the P2X7R^25^, which was only slightly increased by Aβ stimulation. Nimodipine caused a significant drop of mitochondrial potential that was further collapsed by FCCP. Aβ treatment also caused a large increase in mitochondrial potential in primary wt microglia, which was largely inhibited by nimodipine (Fig. 5a,b). Microglia isolated from P2X7R-deleted mice, similarly to N13R cells, showed a very thin mitochondrial network and small (but statistically significant) mitochondrial response to Aβ stimulation (Fig. 6a,b). Aβ is known to promote mitochondrial ROS production^26^. Supplementary Fig. S5 shows that Aβ triggered a small but statistically significant increase in ROS production in N13 microglia. Expression of the P2X7R was also required since N13R cells showed no increase in ROS generation following Aβ stimulation. As a control, we tested ATP that caused a very large, P2X7R-dependent, ROS generation.

The increase of mitochondrial potential due to short-term Aβ stimulation, albeit unanticipated, suggested that ATP synthesis might be enhanced under these conditions as in principle hyperpolarization is suggestive of a tighter mitochondrial coupling and therefore more efficient oxidative phosphorylation. We initially analysed mitochondrial respiratory parameters in microglial cells with the Seahorse apparatus but we were consistently unable to obtain reproducible results in the presence of the amyloid peptide. Therefore, we simply measured total intracellular ATP content in microglia cultures after stimulation with Aβ (Supplementary Fig. S6). Incubation in the presence of Aβ for 5 and 24 h caused a significant decrease in total cellular ATP content, which was paralleled by a modest increase in LDH release, a gross index of cell injury. Incubation in the presence of nimodipine partially rescued the ATP drop and completely prevented LDH release.

**Figure 7:**
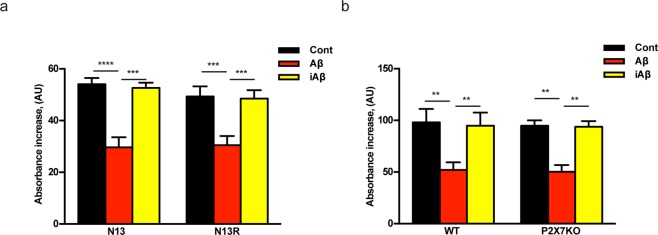
Aβ inhibits F0F1 ATP synthase activity in isolated and permeabilized mitochondria. (**a**) mitochondria isolated from wt N13 or N13R cells; (**b**) liver mitochondria isolated from P2X7R wt or P2X7R-KO mice (see Methods). Mitochondria were suspended at a concentration of 5–10 mg/mL, permeabilized by two rounds of freeze/thawing, exposed to Aβ or iAβ at a concentration of 2 μM, and assayed for ATP synthase activity as described in Methods. Data are means + SEM from 3 independent experiments, each performed in triplicate for a total of 9 individual determinations. **p < 0.01; ***p < 0.001; ****p < 0.000.1.

The finding that Aβ treatment hyperpolarized the mitochondria and at the same time decreased cellular ATP content is intriguing as it is anticipated that mitochondria with a higher membrane potential should synthesize more ATP. However, it is also known that the mitochondrial poison oligomycin, a selective blocker of the mitochondrial F0/F1 ATP synthase^27^ causes at the same time hyperpolarization of mitochondrial potential and inhibition of ATP synthesis. Thus we hypothesized that Aβ might have an oligomycin-like activity. To test this hypothesis we measured F0F1 activity in isolated mitochondria. We isolated a sufficient amount of mitochondria from wt N13 or N13R cells, but we found it very difficult to obtain a sufficient amount of primary microglia for mitochondria isolation, especially from P2X7R-KO pups. Therefore, to allow recovery of an amount of F0F1 complex sufficient to carry out biochemical analysis, we isolated mitochondria from livers of P2X7R wt and P2X7R-KO mice besides wt N13 and N13R cells (Fig. 7). Mitochondria were permeabilized to freely access the F0F1 catalytic site and uncouple the ATP synthase from the respiratory chain. Incubation of isolated mitochondria in the presence of Aβ strongly inhibited ATP synthase activity in both wt N13 and N13R microglia. Interestingly, mitochondria isolated from N13R cells or P2X7R-KO liver were as sensitive to the inhibitory effect of Aβ as those isolated from wt N13 cells or P2X7R wt liver. The scrambled iAβ peptide had no effect. Nimodipine was unable to prevent Aβ-dependent changes in isolated mitochondria. These and our previous data^9^ suggest that interference with mitochondrial energy metabolism might be one of the mechanisms by which Aβ impairs microglia responses.

**Figure 8:**
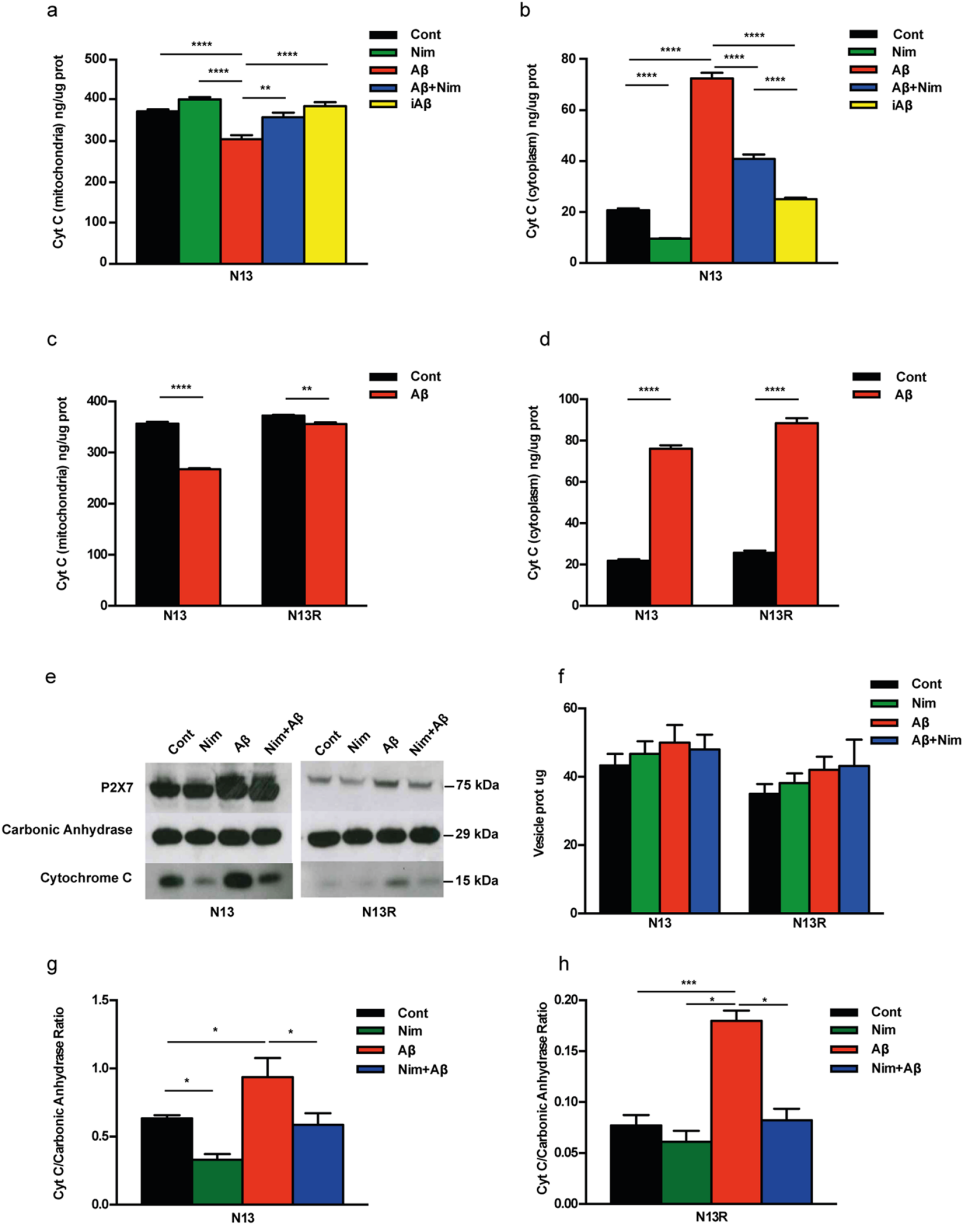
Aβ triggers cyt c release from mitochondria in N13 cells. (**a**–**d**) Microglia was suspended in complete RPMI medium, plated in 10 cm Petri dishes at a concentration of 10^6^/dish for 24 h at 37 °C, and challenged with the various stimuli. Citochrome c (cyt c) was analyzed as described in Methods. (**e**–**h**) Microglia cells were maintained in FCS-supplemented RPMI at for 24 h 37 °C at a concentration of 5 × 10^5^/dish, then the various stimulants were added at the concentration reported in Fig. 1. At the end, supernatants were withdrawn and microvesicles collected as detailed in Methods. Microvesicle suspensions were concentrated to 0.5–1 mg/mL, loaded onto SDS-PAGE and probed for P2X7R and cyt c content. Carbonic anhydrase (1 μg) was exogenously added to the microvesicle suspensions and used as loading standard. Strips were cut from the same blot and probed with the relevant antibodies against the P2X7R, carbonic anhydrase or cytochrome c. (**f**) Microvesicle release from N13 and N13R cells (μg/5 × 10^5^ cells). (**g**,**h**) Densitometric analysis of microvesicle cyt c content normalized on the carbonic anhydrase. Data from (**a**–**d**) are means ± SEM from 3 to 4 independent experiments, each performed in sixtuplicates for a total of 18 to 24 individual determinations. Data from (**f**–**h**) are means ± SEM from to 4 independent experiments. Blot shown in (**e**) is exemplificative of 4 similar. *p < 0.05; **p < 0.01; ***p < 0.001; ****p < 0.000.1.

It is known that Aβ-mediated mitochondrial toxicity is heralded by cytochrome c (cyt c) release^28^, a key event in apoptosis, therefore we asked whether this response was triggered by Aβ in microglia, and possibly inhibited by nimodipine. As shown in Fig. 8a,b, nimodipine by itself slightly but significantly prevented basal cyt c release from the mitochondria and in parallel reduced its accumulation into the cytosol of resting wt N13 microglia. Aβ caused a strong stimulation of cyt c release from the mitochondria and a large accumulation in the cytosol, which were both prevented by nimodipine. The scrambled iAβ peptide had no effect. In the N13R cells, Aβ-triggered an about 30% lower cyt c release compared to wt N13 microglia (Fig. 8c). In both wt N13 and N13R cells in parallel with release from mitochondria, cyt c accumulated into the cytoplasm (Fig. 8d), to a slightly lower level in wt N13 microglia. This was surprising since we anticipated that cytoplasmic cyt c should be higher in wt N13 versus N13R cells since the amount of cyt c released from the mitochondria in wt N13 was about 30% higher than in N13R cells (Fig. 8c). Thus, we asked if some of the cyt c released was removed from the cytoplasm in wt N13 but not in N13R microglia.

Since activated microglia is known to release plasma membrane-derived microvesicles^29^, we hypothesized that cytoplasmic cyt c might be trapped and released via these particles. Figure 8e shows that stimulation of wt N13 or N13R cells with Aβ triggered release of microvesicles that could be collected in the cell supernatants according to current procedures^29,30^. Although P2X7R stimulation is known to trigger a large release of plasma membrane-derived microvesicles from microglia as well other cell types^29,30^, Aβ-driven microvesicle shedding was marginally affected by lack of P2X7R (Fig. 8f). Shed microvesicles are known to contain a plethora of cytoplasmic or membrane-associated molecules, such as NLRP3 inflammasome components, MHC-I and-II molecules, metalloproteases, as well as ATP receptors (e.g. P2X7R itself)^29,30^. Data from Fig. 8e show that microvesicles also contained cyt c. Although a precise estimate of the amount of cyt c contained in the microvesicles is not possible, our data strongly suggest that the lower content of cytoplasmic cyt c in wt N13 versus N13R cells depended on cyt c being trapped within shed microvesicles in wt N13 but not N13R microglia, thus resulting in an amount of microvesicle-associated cyt c many fold lower in N13R versus wt N13 microglia (Fig. 8g,h). Note that values on the y axis in Fig. 8h are ten times lower than in Fig. 8g. Microvesicles released from N13R cells also had a very low P2X7R content (Fig. 8e). Overall microvesicles release was similar in N13R and wt N13 cells. Thus, data in Fig. 8 show that stimulation of microglia cells with Aβ triggered a large release of cyt c from the mitochondria, which was in part sequestered within shed microvesicles. Sequestration of cyt c within the microvesicles only occurred in the presence of the P2X7R. Nimodipine had no effect on microvesicle release whether in the absence or presence of Aβ, but drastically reduced basal and Aβ-stimulated microvesicle cyt c content (Fig. 8e).

It is known that P2X7R may promote phagocytosis in microglia^31^ and ATP stimulation promotes pinocytosis^32^, thus we investigated whether the protective effect afforded by P2X7R deletion might depend on a reduced pinocytic uptake of Aβ, and whether this might also explain the protective effect of nimodipine. As shown in Supplementary Fig S7 N13R cells did show a reduced pinocytic uptake of Texas red-labelled ovalbumin, but this is unlikely to explain the protective effect of nimodipine as in the presence of this drug pinocytosis was basically unaffected.

## Discussion

Despite the enormous expenditure of efforts and financial resources in the investigation of Alzheimer’s disease, payback in terms of novel efficacious drugs has been highly disappointing^1^. This might be one of the reasons behind the recent decision of a major Pharmaceutical Company such as Pfizer to withdraw from Alzheimer’s research^33^. Such years-long and deluding search suggests that identification of novel cellular targets, intracellular pathways and candidate pharmacological compounds is absolutely needed. Identification of the molecular mechanism of Aβ-dependent toxicity is a hot focus in Alzheimer’s research. This misfolded protein is understood to have both a direct neuronal cell-centred effect and a broader microglia-targeted pro-inflammatory activity^34^. Accruing observations show that mitochondrial dysfunction is often found in Alzheimer’s patients, thus raising the issue of an Aβ-mediated impairment of mitochondrial oxidative phosphorylation^35–37^. Previous studies focused on alterations in mitochondrial respiratory complexes, mainly complex IV (cytochrome c oxidase), the mitochondrial permeability transition pore (mPTP)^38^, and more recently on the F0F1 ATP synthase^37^. In this latter study, mitochondria localized at neuronal synapsis were found to be exquisitely sensitive to Aβ-mediated damage due to selective removal of the oligomycin sensitivity-conferring protein (OSCP) of the ATP synthase complex^37^. Aβ accumulates in mitochondria of neuronal cells very likely via the translocase of outer membrane (TOM) and putatively localizes to mitochondrial christae^39^. It is not known whether mitochondrial toxicity might also underlie Aβ-mediated injury of non-neuronal cells.

Microglia is now generally acknowledged to be an additional important target of Aβ-dependent toxicity, but the pathogenic mechanisms are far from clear. In fact, microglia has a dual effect on AD pathogenesis, a detrimental activity dependent on release of neurotoxic factors such as ROS, cytokines, chemokines or metalloproteases, and a protective function based on microglia ability to remove Aβ or accelerate its degradation^34,40^. However, it is increasingly evident that in AD microglia is unable to fulfil its phagocytic tasks, but the mechanism underlying this dysfunction is unknown^41^. In the present study, we show that in mouse microglia Aβ severely hampered mitochondrial energy metabolism by targeting the F0F1 ATP synthase, thus resulting in 50% reduction of activity and in about 30% decrease of total ATP content over a 24 h incubation. Interestingly, Aβ treatment did not significantly affect the function of the respiratory chain, but rather caused hyperpolarization of the mitochondria, as it would be anticipated on the basis of an oligomycin-like effect. Despite the undisputed contribution to phagocytosis of anaerobic glycolysis, it is clear since the very early studies by Cohn^32^ that oxidative metabolism is also required, thus mitochondrial dysfunction could seriously impair removal of extracellular particulate matter. Therefore, we think that mitochondrial dysfunction triggered by Aβ causes a complex dysregulation of microglia functions characterized on one hand by an acute stimulation of pro-inflammatory cytokine release, and on the other on a long term reduction of phagocytic activity, and therefore of removal of extracellular Aβ fibrils. However, Aβ needs the cooperation of an additional partner to successfully injure microglia: the P2X7R.

We previously showed that in mouse microglia Aβ-dependent release of the pro-inflammatory cytokine IL-1β is strictly dependent on expression of a functional P2X7R, likely due to direct interaction of the amyloid peptide with this receptor^15^. Here we further extend these early observations by showing that Aβ-dependent NLRP3 gene transcription and NLRP3 inflammasome stimulation were also P2X7R-mediated. Thus, in microglia the whole Aβ-stimulated, inflammasome-associated, pro-inflammatory machinery was P2X7R-dependent. Even more interestingly, our findings show that also mitochondrial dysfunction in microglia was P2X7R dependent. N13R cells or primary microglia lacking the P2X7R were resistant to Aβ-dependent mitochondrial hyperpolarization. In addition, N13R cells were also resistant to Aβ-triggered cyt c release, another event heralding mitochondrial toxicity.

Based on our previous study suggesting a direct interaction of Aβ with the P2X7R^15^, we hypothesize that the P2X7R facilitates Aβ cellular uptake and mitochondrial localization, and therefore is permissive for mitochondrial injury. The P2X7R is reported to have scavenger activity independent of its pore-forming function, possibly relevant in the central nervous system^42,43^, and to promote endocytosis of extracellular anti-bactetial peptides (e.g. LL-37)^44^. Extracellular ATP is long known to stimulate pinocytosis^32^, and more recently basal activity of the P2X7R was shown to promote innate phagocytosis^45^. Thus, it is conceivable that the facilitating role of the P2X7R in supporting Aβ-dependent toxicity resides in its promotion of pinocytosis or innate phagocytosis, as shown in Suppl Fig S7. In support of this view, lack of the P2X7R did not prevent Aβ inhibition of F1/F0 ATP synthase activity in isolated and permeabilized mitochondria, showing that once the mitochondria are freely accessible to Aβ lack of the P2X7R has no protective effect, nor there is an intrinsic P2X7R-associated F1/F0 ATP synthase defect.

Search for effective therapies for AD has been substantially fruitless. This failure underscores the need to identify novel targets and drugs. A few year ago we reported that the well-known voltage-gated Ca^2+^ channel blockers nimodipine and nitrendipine fully inhibited Aβ-stimulated IL-1β release from mouse microglia^9^. Since brain microglia does not express functional L-type Ca^2+^ channels, we hypothesized off-target effects of the dihydropyridine compounds. Data shown in the present study support this hypothesis as nimodipine strongly reduced all Aβ-dependent effects, i.e. NFκB activation, NLRP3 inflammasome stimulation, IL-1β release, mitochondrial dysfunction and LDH and cyt c release. Other members of the dihydropyridine family, i.e. nifedipine and nitrendipine, also inhibited Aβ-stimulated IL-1β release, and like nimodipine had no effect on Aβ stimulated TNFα release. We don’t think that nimodipine directly antagonizes the P2X7R since early responses such as Ca^2+^ influx and uptake of extracellular fluorescent markers are unaffected^9^, but we rather believe that it targets a pathway downhill to the P2X7R, although we have no evidence so far of the molecular identity of this pathway. Mitochondria are an attractive target, although the finding that nimodipine had no protective effect on isolated mitochondria speaks against a direct protective effects towards Aβ-mediated inhibition of F0F1 ATP synthase. More likely, nimodipine prevented delivery of Aβ to the mitochondria. However, this protective effect is not due to inhibition of pinocytic uptake as nimodipine causes, if any think, a modest increase in pinocytosis. Thus, we hypothesize that nimodipine might accelerate endosome-lysosome fusion, and therefore Aβ degradation, but at the moment we have no proof for this. Here we show that, whichever the pathway by which Aβ reaches the mitochondria, mitochondrial dysregulation is at basis of the wide-range activation of inflammatory responses, i.e. NFκB activation, NLRP3 expression, casp-1 activation and IL-1β maturation and release^46^. Thus, nimodipine by preventing Aβ-dependent mitochondrial toxicity, prevents the activation of several key pro-inflammatory pathways.

In summary, this study highlights a novel pathway for the inhibition of microglia cell functions by Aβ, further underlines the key role of the P2X7R as a permissive factor for Aβ toxicity in mouse microglia, and demonstrates the potent protective effect of nimodipine. We showed previously that nimodipine prevents *in vivo* intra-hyppocampal accumulation of IL-1β triggered by Aβ inoculation^9^, thus nimodipine administration might be a viable therapeutic strategy for AD.

## Methods

### Cells

Microglial N13 cells were grown in RPMI-1640 medium, heretofore referred to as RPMI medium, supplemented with 2 mM glutamine, 10% fetal calf serum (FCS) (Gifco BRL, Basel, Switzerland), 100 U/ mL, penicillin and 100 mg/mL streptomycin. Primary mouse microglia cells were isolated from 2 to 4-day-old post-natal mice as described previously^15^. More than 98% of cells were identified as microglia using a macrophage cell-specific F4/80 biotinylated mAb antibody (Serotec, Dusseldorf, Germany) followed by staining with Oregon Green 488 goat anti-rat IgG (Molecular Probes, Leiden, The Netherlands). All animal care and experimental procedures complied with institutional and national guidelines (see below). Microglia were plated in astrocyte-conditioned medium (high glucose-DMEM supplemented with 2 mM glutaMAX™ (Gibco Life Technologies Europe BV, Monza, Italy), 10% FCS, 100 U/mL penicillin and 100 mg/mL streptomycin), and used for experiments 24 h after plating. Short-term experiments were run either in FCS-free RPMI medium or in a saline solution with the following composition: 125 mM NaCl, 5 mM KCl, 1 mM MgSO_4_, 1 mM NaH_2_PO_4_, 20 mM HEPES, 5.5 mM glucose, 5 mM NaHCO_3_, 1 mM CaCl_2_, pH 7.4. Nimodipine, nitrendipine and nifedipine (Sigma-Aldrich Srl, Milano, Italy), were dissolved in dimethyl sulfoxide at a concentration of 10 mg/mL. Long-term (chronic) experiments were performed in FCS-supplemented RPMI medium (complete RPMI) (wt N13 and N13R cells), or in astrocyte-conditioned medium (primary microglia). All experiments were performed at 37 °C. Lipopolysaccharide (LPS) from Escherichia coli serotype 055:B5 (Sigma-Aldrich) was dissolved in PBS at a concentration of 1 mg/ml. Aβ 1–42 and the inactive scrambled Aβ peptide (iAb) 42–1 were purchased from Bachem (Bubendorf, Switzerland). Aβ peptides were dissolved in dimethyl sulfoxide at a concentration of 1 mM. Freshly prepared Aβ solution used in the experiments (final concentrations from 4 to 10 μM) mainly consists of monomers/oligomers, as witnessed by staining (Supplementary Fig S8).

### Animals

Procedures involving animals and their care were conducted in conformity with the institutional guidelines that are in compliance with national (D.lgs. n. 26/2014), and international laws and policies (EU Directive 86/609/EEC; Guide for the Care and Use of Laboratory Animals, U.S. National Research Council, 1996), and were approved by the Italian Ministry of Health (authorizations n. 75/2013 released on 25/03/2013, 716/2016-PR released on 18/05/2016, and 744/2018-PR released on 01/09/2018).

All efforts were made to minimize the number of animals used and their suffering. The results of all studies involving animals are reported in accordance with the ARRIVE guidelines for reporting experiments involving animals. *Ex vivo* samples used in this study were from a total of 32 C57Bl/6 wt (Envigo-Harlan Laboratories, Udine, Italy) or *P2rx7*-KO female mice (kind gift of GlaxoSmithKlein, UK) weighing 18–20 g. They were housed in the animal facility of the University of Ferrara at constant room temperature (22 ± 1 °C) and relative humidity (55 ± 5%) under a 12 h light/dark cycle (lights on from 7:00 AM to 7:00 PM). Food and water were freely available.

### Isolation of mitochondria

Mitochondria from wt N13 or N13R cell cultures or from mouse livers were isolated as described by Wieckowski *et al*.^47^. For isolation from livers, 6-week old C57Bl/6 (male) mice were sacrificed by cervical dislocation, livers extracted and minced at 4 °C in about 10 volumes of BSA-containing mannose-sucrose-hepes-EDTA (MSHE) buffer^47^, and rinsed several times to remove blood. All following procedures were performed at 4 °C. Tissue was homogenised by applying 10 strokes of a drill-driven Teflon glass homogenizer. Homogenate was centrifuged at 800 g for 10 min, fat/lipid contamination was removed by careful aspiration, and the remaining supernatant was centrifuged at 10000 g for 10 min. After removal of the light mitochondrial layer, pellet was suspended in BSA-containing MSHE, and centrifuged one more time at the same speed. The final pellet was suspended in a minimal volume of BSA-containing MSHE. Total protein concentration (mg/mL) was determined by the Bradford method (Bio-Rad Laboratories, Segrate, Milano, Italy). Typically, an amount of about 7.5 mg of mitochondria was obtained from a single mouse liver.

### Measurement of IL-1β, TNFα and of enzymatic activity

Lactate dehydrogenase (LDH) activity was measured according to standard laboratory procedures. IL-1β was measured with a R&D kit (R&D, Minneapolis, MN, USA). TNFα was measured with a Quantikine ELISA kit (R&D). F0F1 ATP synthase activity was measured by a coupled enzyme assay in isolated mitochondria permeabilized with two rounds of freeze/thawing, and suspended in phenol red and serum-free RPMI. Briefly, mitochondria were incubated in RPMI at a concentration of 30 μg/mL in a spectrophotometer cuvette in the presence of pyruvate kinase (0.8 U/mL), lactate dehydrogenase (1.12 U/mL), phosphoenol pyruvate (1.5 mM), antimycin A (1 μM), rotenone (1 μM), ATP (200 μM) and NADH (100 μM). ATP is hydrolysed by F0F1 ATP synthase to produce ADP that is used by pyruvate kinase to generate pyruvate at the expenses of phosphoenolpyruvate, thus regenerating ATP. Pyruvate is oxidized to lactate by lactate dehydrogenase (LDH) in the presence of NADH, that is in turn oxidized to NAD^+^. NADH oxidation is measured by spectrophotometry at 340 nm. F0F1 ATP synthase-independent NADH oxidation rate was measured in the presence of oligomycin (2 μM), and subtracted from total oxidation rate.

### Measurement of NFκB activity

NFκB activity was assessed by measuring nuclear translocation of the p65-NFκB subunit with the Nuclear Extraction Kit (Active Motif, Carlsbad, CA, USA) and the NFκB transcription Factor Assay Kit (Abcam, Cambridge, UK). Briefly, after treatment with various stimulants, cells were lysed and processed according to manufacturer’s instructions. Supernatants were collected and transferred to 96-well plates coated with a specific double stranded DNA (dsDNA) sequence containing the p65-NF-κB response element. p65-NF-κB was detected by addition of specific primary antibody, and visualized by ELISA.

### Western blotting

Cells were detached by scraping and lysed in lysis buffer (300 μM sucrose, 1 mM K_2_HPO_4_, 1 mM MgSO_4_, 5.5 mM glucose, 20 mM HEPES (pH 7.4), 1 mM benzamidine, 1 mM phenylmethylsulfonyl fluoride, 0.2 μg DNase, and 0.3 μg RNase, all by Sigma-Aldrich) by repeated freeze/thawing cycles. Proteins were separated on Novex NuPage Bis-Tris 4–12% precast gel (Life Technologies, Milano, Italy) and transferred to nitrocellulose membranes (GE Healthcare-Life Sciences, Milano, Italy). After incubation with TBS–Tween-20 (0.1%) supplemented with 2.5% non-fat powdered milk plus 0.5% BSA for 1 h to saturate unspecific binding sites, membranes were incubated overnight with primary antibodies at 4 °C. The anti-NLRP3 polyclonal antibody (Imgenex, Bio-Techne Srl, Milano, Italy, cat. N. NBP2-12446) was diluted 1:500. The anti-actin polyclonal antibody (Sigma-Aldrich, cat. N. A5060) was diluted 1:1,000. The anti-P2X7R polyclonal antibody (Merck-Millipore, Milano, Italy, cat n. AB5246) was diluted 1:1,000. The anti-cytochrome C monoclonal antibody (cat. n. Ab 13575, Abcam) was diluted 1:1,000 and the anti- carbonic anhydrase II (CA2) polyclonal antibody (Sigma-Aldrich, cat. n. SAB 2900749) was diluted 1:1000. Membranes were incubated with secondary goat anti-rabbit HRP-conjugated antibodies (Invitrogen-Thermo Fisher Scientific, Monza, Italy, cat n. 31460) at a 1:3,000 dilution for 1 h at room temperature. Densitometric analysis of the protein bands was performed with the freely available ImageJ software. Band density was normalized over the actin band. All the antibody were diluted in TBS–Tween-20 (0.1%) supplemented with 2.5% non-fat powdered milk plus 0.5% BSA.

### Intracellular Ca^2+^ measurement

Cytoplasmic Ca^2+^ concentration was measured at the wavelength excitation 340/380 and emission 505 with the fluorescent indicator fura-2/AM (Thermo Fisher Scientific) in a thermostat-controlled (37 °C) and magnetically-stirred Cary Eclipse Fluorescence Spectrophotometer (Agilent Technologies, Milano, Italy). Cells were loaded with 4 μM fura-2/AM in the following saline solution: 125 mM NaCl, 5 mM KCl, 1 mM MgSO_4_, 1 mM NaH_2_PO_4_, 20 mM HEPES, 5.5 mM glucose, 1 mM CaCl_2_, pH 7.4, supplemented with 250 μM sulfinpyrazone. After loading, cells were rinsed and re-suspended at a concentration of 10^6^/ml in the above saline solution.

### Pinocytosis

Pinocytosis was measured by Texas red-albumin uptake. Briefly, N13 or N13R cells were incubated in the presence of a concentration of 30 μg/ml of ovalbumin Texas red conjugate (Thermo Fisher Scientific) for 1 hour in the presence of absence of nimodipine, rinsed and then cell-associated fluorescence was measured in a Wallac Victor Perkin Elmer (Perkin Elmer, Beaconsfield, UK) plate reader. Cells were also analysed by microscopy to verify intracellular formation of pinocytic vacuoles.

### Microscopy

Microscopy was performed with a temperature-controlled Zeiss LSM 510 confocal microscope (Carl Zeiss, Arese, Italy). Mitochondrial potential was measured by monitoring uptake of the positively-charged dye TMRM as previously described^25^. Reactive oxygen species were detected using the fluorogenic CellROX™ 488 reagent (Invitrogen), according to manufacturer’s instructions. Briefly, N13 cells were seeded on 24-well plates and challenged with the different stimuli. After a 1 h incubation, the dye was then added to at a concentration of 10 µM and fluorescence measured by fluorescence microscopy at an excitation wavelength of 488 nm.

### Measurement of extracellular ATP

ATP was measured in cell lysates with the rluciferase/luciferin method (Enliten rluciferase/luciferin, Promega, Italy) in a Firezyme luminometer (Biomedica Diagnostics Inc, Windsor, Canada). Data are expressed as relative luminescence units (RLU), and converted into nanomoles of ATP thanks to a calibration curve performed by adding known ATP amounts.

### Microvesicle preparation

Cells were plated in 6-well culture dishes in the absence or presence of the various stimulants at a concentration of 5 × 10^5^/well. Then, supernatants were withdrawn and centrifuged at 800 × g for 10 min to get rid of cells and cell debris. The supernatant was further centrifuged at 10,000 × g, and the pellet (microvesicles) lysed in a 300 mM sucrose solution supplemented with 0.1 Triton X-100 plus PMSF and benzamidine. Centrifugations and further manipulations were performed at 4 °C.

### Statistical analysis

All data are shown as means ± standard error of the mean (SEM). Test of significance was performed by two-way ANOVA using GraphPad InStat software (GraphPad, San Diego, Ca, USA).

## Supplementary information

Amyloid β-dependent mitochondrial toxicity in mouse microglia requires P2X7 receptor expression and is prevented by nimodipine

## Data Availability

The datasets generated during and/or analyzed during the current study are available from the corresponding author on reasonable request.
