# Supplementary material for: Amyloid β-dependent mitochondrial toxicity in mouse microglia requires P2X7 receptor expression and is prevented by nimodipine
Source: Sci Rep. 2019 Apr 24;9:6475. doi: 10.1038/s41598-019-42931-2 (PMC6482182; doi:10.1038/s41598-019-42931-2)
Supplement: Supplementary file 1 — Amyloid β-dependent mitochondrial toxicity in mouse microglia requires P2X7 receptor expression and is prevented by nimodipine [file 41598_2019_42931_MOESM1_ESM.pdf]

Supplementary information

Amyloid β-dependent mitochondrial toxicity in mouse microglia requires P2X7 receptor expression and is prevented by nimodipine

Paola Chiozzi, Alba Clara Sarti, Juana M. Sanz, Anna Lisa Giuliani, Elena Adinolfi, Valentina Vultaggio-Poma, Simonetta Falzoni and Francesco Di Virgilio

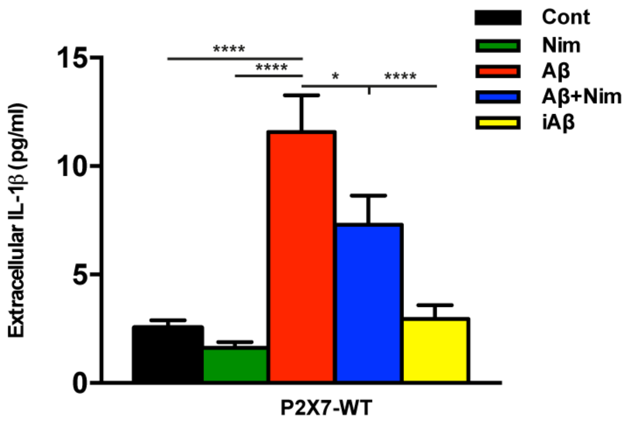

Supplementary Figure S1. Aβ triggers IL-1β secretion. Primary mouse microglia, suspended in 200 µl of FCS-supplemented astrocyte-conditioned medium was plated in 48-well culture dishes at a concentration of $2x10^4$/well at 37°C for 24h. At the end, supernatants were withdrawn and analysed for IL-1β content. Stimulants concentration was: Aβ, 4 µM; iAβ, 4 µM; nimodipine (nim), 36 nM. Data are means ± SEM from 3 to 5 independent experiments, each performed in triplicate, for a total of 9 to 15 individual determinations. *p < 0.05; ****, p > 0.0001.

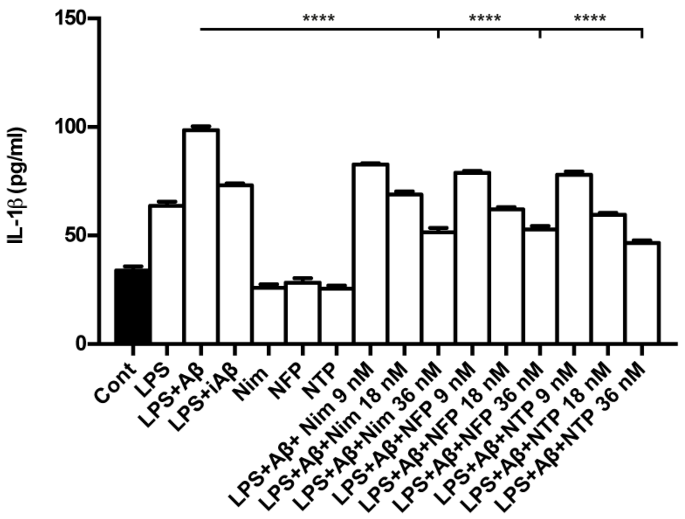

Supplementary Figure S2. Inhibition of Aβ-triggered IL-1β release by increasing concentrations of nimodipine, nifedipine, and nitrendipine. N13 microglial cells were plated at a concentration of $5x10^4$/well in a 48-well plate and pretreated for 4 h with LPS (1 mg/ml) in 10% FCS-supplemented RPMI medium. After this time, RPMI was withdrawn and replaced with a saline solution containing 125 mM NaCl, 5 mM KCl, 1 mM $MgSO_4$, 1 mM NaH_2PO_4, 20mM HEPES, 5.5 mM glucose, 5 mM $NaHCO_3$, 1 mM $CaCl_2$, pH 7.4 (final volume 150 µl), also supplemented with Aβ or iAβ (both 10 µM), in the presence of increasing concentrations of nimodipine (nim), nifedipine (NFP), or nitrendipine (NTP). Incubation was carried out under these conditions for further 30 min. Controls received no treatment. Data are averages ± SD on quadruplicate samples for each condition. ****, p<0.0001.

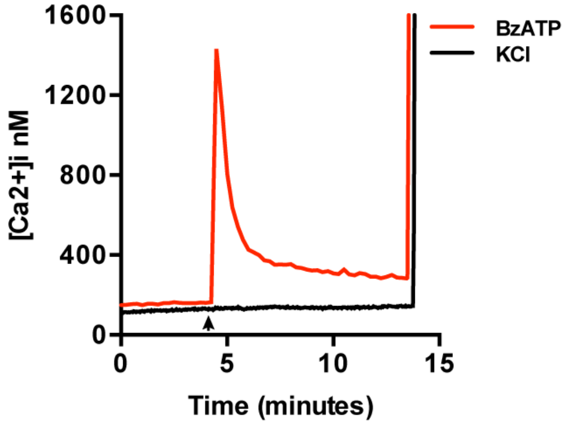

Supplementary Figure S3. Plasma membrane depolarization does not trigger an intracellular Ca^{2+} rise in N13 microglia. Fura-2-loaded N13 microglial cells were incubated at a concentration of 10^6/ml in the fluorimeter cuvette and stimulated (arrow) with 50 mM KCl (black trace) or 300 µM BzATP. At the end of each trace 100 nM ionomycin was also added to trigger a maximal Ca^{2+} response. These traces are exemplificative of four obtained in different experiments and with different cell preparations. In no instance a KCl-induced increment in the cytosolic Ca^{2+} concentration was observed.

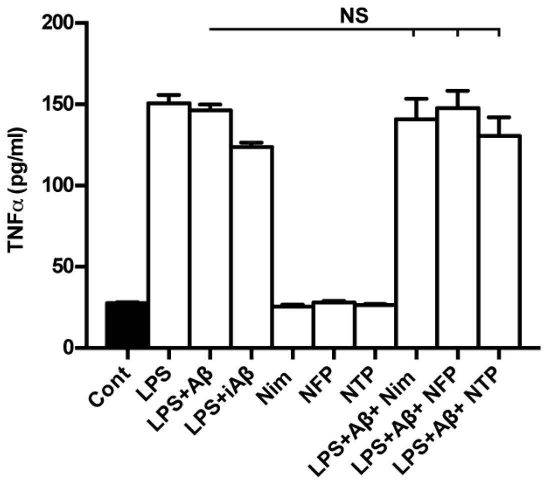

Supplementary Fig. S4. Dihydropyridines have no effect of LPS and Aβ-stimulated TNFα release from N13 microglia. N13 cells were incubated in the presence of the various stimuli and inhibitors as described in Supplementary Fig.S2. Data are averages ± SD of quadruplicate samples.

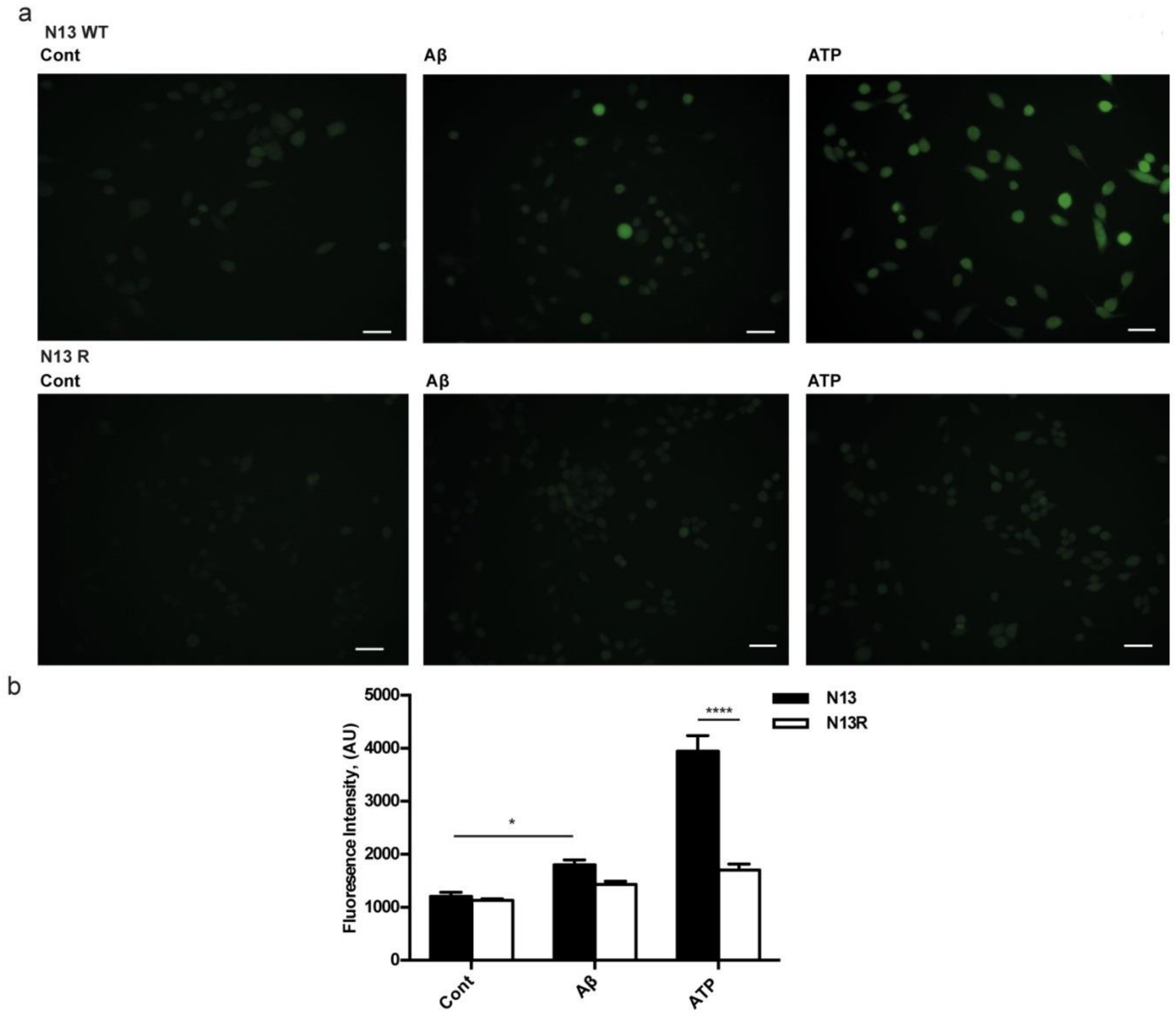

Supplementary Figure S5. ATP and Aβ stimulate ROS production in N13, but not in N13R cells. N13 and N13R cells were plated in 24-well culture dishes at a concentration of 1.5 x10^5/well and treated with the indicated stimuli at 37°C for 1 h (Aβ 4 μM, ATP 500 μM). For assessment of ROS production, cells were stained with CellROX, and analyzed by microscopy. (**a**) fluorescence microscopy pictures; bar = 30 μm. (**b**) mean fluorescence emission ± SEM from 3 independent experiments, each performed in triplicate for a total of 9 individual determinations. *, p>0.05; ****p < 0.0001.

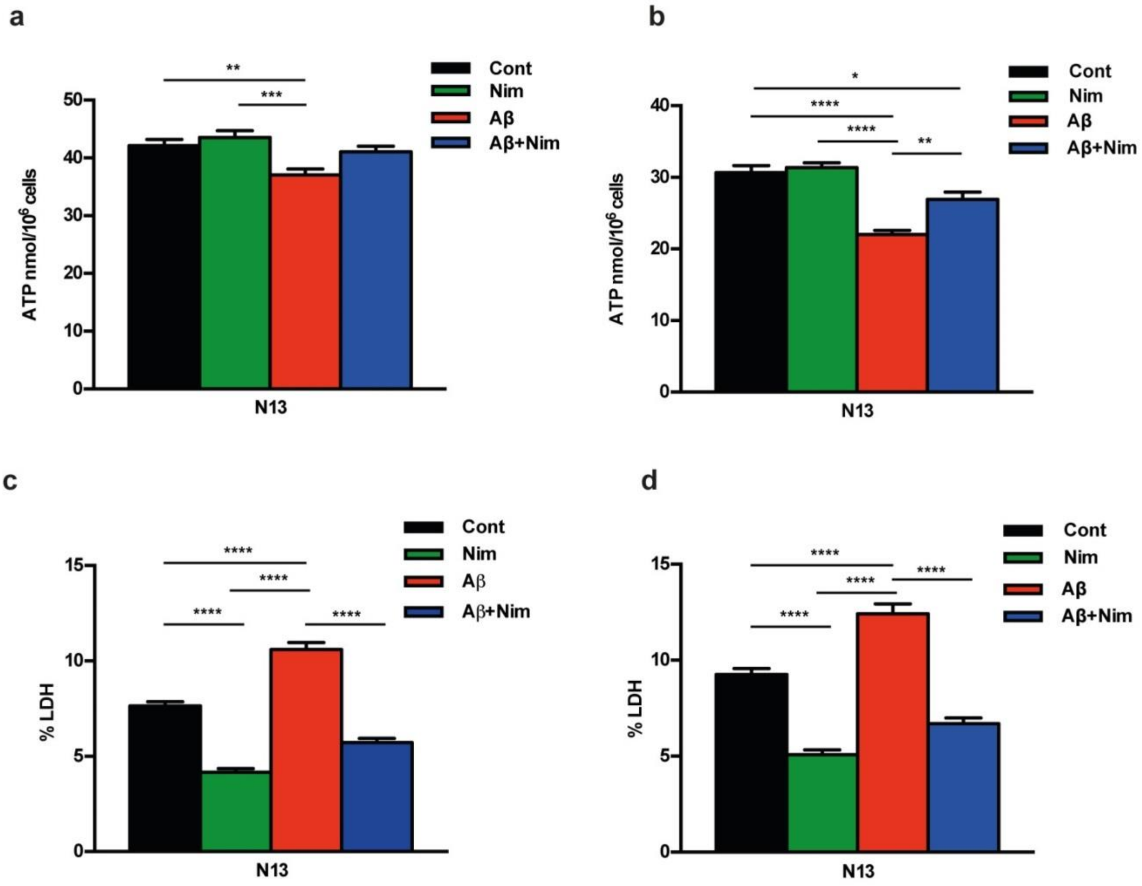

Supplementary Figure S6. Aβ decreases total intracellular ATP content and increases lactic dehydrogenase (LDH) release. For LDH determinations, cells were re-suspended in FCS-supplemented RPMI medium and plated in 24/well plates at a concentration of 1.5 $x10^5$/well (5 h incubation, **a,c**) or $7.5x10^4$/well (24 h incubation, **b,d**). For ATP determinations, cells were plated in complete RPMI for 5 h or 24 h at 37°C in luminometer-dedicated micro-well plates (FireZyme) as described in Methods, at a concentration of 1000 (5 h) or 500 (24 h) cells/well. At the end, pellets (**a-b**) or supernatants (**c-d**) were withdrawn and analyzed for ATP (**a-b**) or LDH (**c-d**) content. Stimulant concentration as in Figure 1. Data are means ± SEM from 3 independent experiments, each performed in triplicate for a total of 9 individual determinations. *, $p < 0.05$; **, $p < 0.01$; ***, $p < 0.001$; ****$p < 0.0001$.

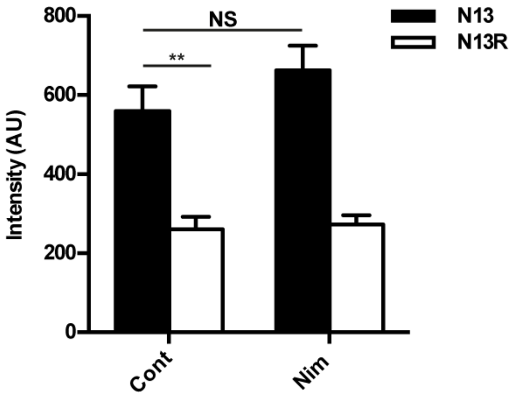

Supplementary Figure S7. P2X7R deletion impairs pinocytosis. Lack of effect of nimodipine. N13 microglial cells were seeded at a concentration of $3x10^5$ in 24/well plates in complete RPMI medium, incubated at 37° C for 1 hour in the absence or presence of nimodipine, then Texas red-ovalbumin was added, and the incubation carried out for further 30 min. At the end of this incubation, cells were rinsed three times with PBS, and cell-associated fluorescence measured at the wavelength pair 596/615 nm. Data are averages from three separate experiments each performed in sextuplicate.**$p < 0.01$.

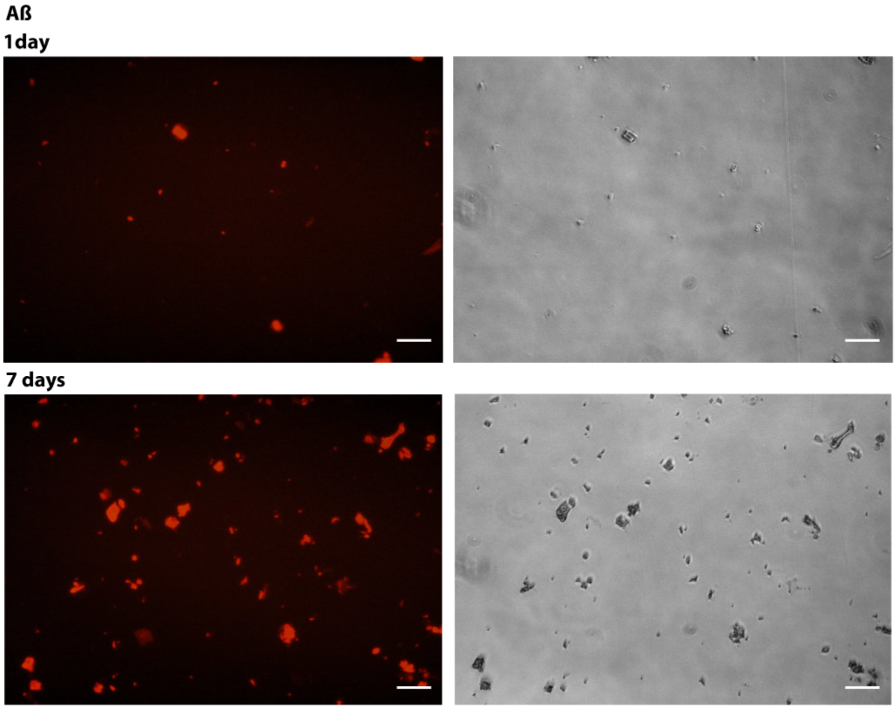

Supplementary Figure S8. Aβ dissolved in PBS was incubated in 96-well plates at a concentration of 10 μM at 37°C for 1 or 7 days. After this time Congo red (10 μg/ml) was added and the incubation carried out for further 10 min before fluorescence or phase pictures were taken with an Olympus IMT-2 microscope (Olympus Optical Co, Tokyo, Japan) equipped with a 20 x objective and a rhodamine filter. Bars = 20 μM.

Figure 2

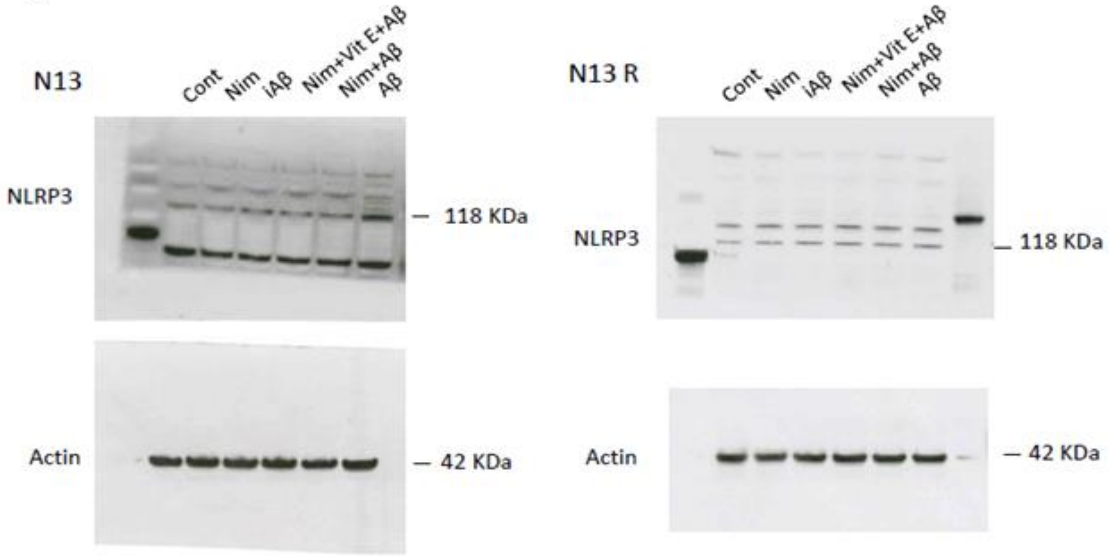

Figure 8

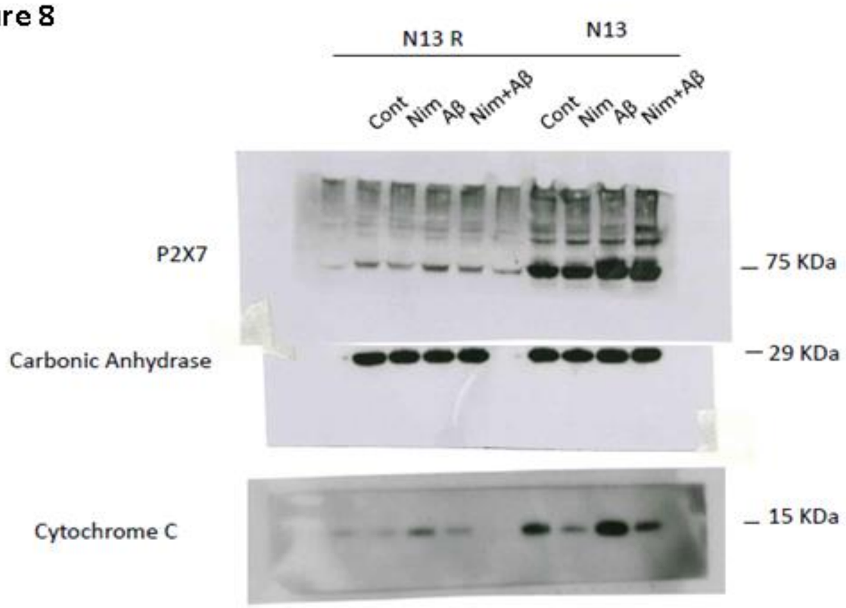

Full length blots related to Figs 2 and 8.
